# Adrenal aldosterone synthase (CYP11B2) histopathology and its association with disease-induced sudden death: a cross-sectional study

**DOI:** 10.1016/j.lanepe.2025.101226

**Published:** 2025-02-06

**Authors:** Antero Ylänen, Juhani Isojärvi, Antti Virtanen, Helena Leijon, Tiina Vesterinen, Aapo L. Aro, Heini Huhtala, Eeva Kokko, Ilkka Pörsti, Marianna Viukari, Pasi I. Nevalainen, Niina Matikainen

**Affiliations:** a Faculty of Medicine and Health Technology, Tampere University, Tampere, Finland; b Department of Internal Medicine, Tampere University Hospital, Tampere, Finland; c Forensic Medicine Unit, Finnish Institute for Health and Welfare, Helsinki, Finland; d HUS Diagnostic Center, HUSLAB, Department of Pathology, Helsinki University Hospital and University of Helsinki, Helsinki, Finland; e Heart and Lung Center, Helsinki University Hospital and University of Helsinki, Helsinki, Finland; f Health Sciences, Faculty of Social Sciences, Tampere University, Tampere, Finland; g Endocrinology, Helsinki University Hospital, ENDO-ERN (European Reference Network on Rare Endocrine Conditions) and University of Helsinki, Helsinki, Finland

**Keywords:** Primary aldosteronism, Sudden death, Aldosterone synthase, CYP11B2, Hypertension, Pheochromocytoma, Histopathological classification, Aldosterone-producing adenoma, Aldosterone-producing nodule, Aldosterone-producing micronodule

## Abstract

**Background:**

Unidentified cardiovascular risk factors may account for approximately half of sudden deaths, a devastating event with limited preventive tools. We investigated whether adrenal histopathology suggestive of primary aldosteronism, pheochromocytoma, or adrenal masses could explain part of the risk for disease-induced sudden death (DSD).

**Methods:**

In this study, autopsies and histopathological analyses, including aldosterone synthase staining of adrenal glands, were performed on 403 consecutive individuals who experienced sudden death. These individuals were classified into 258 cases of DSD and 144 deaths caused by trauma, suicide, or intoxication, i.e., non-disease-induced sudden death (nDSD). This trial was registered at ClinicalTrials.gov (NCT05446779).

**Findings:**

Adrenal histopathology revealed changes in 31 (7.7%) subjects of the cohort. Of these, the most prevalent findings [25 (6.2%)] were aldosterone-producing adenomas (APA) or nodules (APN), which were associated with myocardial infarction and atherosclerosis at autopsy. Individuals in the DSD group and the subgroup with sudden cardiac death (SCD) were more likely to have APA or APN than individuals in the nDSD group [23 (8.9%) vs. 2 (1.4%), p = 0.002; 16 (8.8%) vs. 2 (1.4%), p = 0.003, respectively]. APA or APN were explanatory factors for DSD (odds ratio [OR] 6.47, 95% confidence interval [CI] 1.40–29.88, p = 0.017) and SCD (OR 10.68, 95% CI 2.02–56.43, p = 0.005). Other findings included two pheochromocytomas, one bilateral adrenal metastasis, and two unilateral adrenal metastases.

**Interpretation:**

In this exploratory study, APA or APN were more frequently seen in DSD and SCD than nDSD cases. Whether primary aldosteronism constitutes a novel risk factor for sudden death warrants further study.

**Funding:**

Finnish State Research funds and independent research foundations: 10.13039/100010133Aarne Koskelo Foundation, the Finnish Kidney Foundation, and the 10.13039/501100005633Finnish Foundation for Cardiovascular Research.

**Research in context**

**Evidence before this study**

Sudden death is a high-priority public health concern, affecting at least 249,500 individuals each year in the European Union. Current knowledge of the risk factors for disease-induced sudden death is limited, which prevents the identification of those at high risk. Adrenal diseases, including primary aldosteronism and pheochromocytoma, are linked to arrhythmia, hypertension, and atherosclerosis, but they are underdiagnosed worldwide. Importantly, the specific contribution of these diseases to sudden death remains unknown. Based on our search of PubMed for peer-reviewed papers published from inception to 1 December 2024, using the terms “sudden death” OR “sudden cardiac death” OR “cause of death” AND “primary aldosteronism” OR “hyperaldosteronism” OR “CYP11B2” OR “pheochromocytoma”, only limited information is available. A few case studies have associated primary aldosteronism with lethal arrhythmias, although they lack histopathological confirmation. To our knowledge, no evidence describes the histopathological findings of CYP11B2 immunostaining in the adrenal glands of individuals who have succumbed to disease-induced sudden death.

**Added value of this study**

The cross-sectional data from this study indicate that adrenal histopathology consistent with aldosterone-producing adenomas or nodules is 6.4 times more frequent among those who experienced disease-induced sudden death than among those who died from non-disease-related causes. The finding of an aldosterone-producing adenoma or adrenal nodules and decrease of CYP11B2 continuity indicating potential autonomous aldosterone production was associated with markers of cardiovascular morbidity at autopsy. These histological findings appear to be prominent independent predictors of sudden death. Pheochromocytoma was found in two cases out of 258 who died due to disease-induced sudden death.

**Implications of all the available evidence**

Our exploratory results suggest that aldosterone-producing adenoma or adrenal nodules, and more generally, a reduction of CYP11B2 continuity in the zona glomerulosa, are more frequently observed in the adrenal glands of disease-induced sudden death cases than those of non-disease-induced sudden deaths. In addition, histopathological findings indicative of autonomous aldosterone production were associated with the presence of atherosclerosis. Consistent with previous case reports, two cases of pheochromocytomas were identified in the disease-induced death group. High rates of adrenal pathology, especially CYP11B2-positive findings and possibly pheochromocytomas, could have implications for the prevention of sudden death, as low-cost biochemical screening for primary aldosteronism and pheochromocytoma is available. For the first time, the European Society of Cardiology 2024 guideline recommends considering systematic evaluation for primary aldosteronism in all hypertensive patients at diagnosis, along with increased use of mineralocorticoid receptor antagonists. Whether increased recognition and targeted therapy of primary aldosteronism can reduce the risk of sudden death warrants further investigation.

## Introduction

Sudden death, including disease-induced death and, specifically, sudden cardiac death (SCD), is a major public health burden worldwide with devastating repercussions for individuals and society.^1^^,^^2^ Established cardiovascular (CV) risk factors have been identified in only 38–58% of young SCD patients, indicating a considerable causal role of unidentified CV risk factors.^3^^,^^4^ In addition to age, sex, ethnicity, and socioeconomic status, suggested explanatory biomarkers for sudden death either reflect general CV risk factors or are specific for various underlying disease pathologies in the general population.^5^ In 40% of sudden deaths among young people, the cause remains undetermined even after a comprehensive autopsy and toxicology analysis.^6^ Taken together, the prediction and prevention of sudden death remain challenging due to multiple knowledge gaps.^2^

Adrenal pathology, including primary aldosteronism (PA), hypercortisolism, or pheochromocytoma, is an important cause of secondary hypertension or premature mortality. These disorders require specific treatments but remain largely underdiagnosed.^7^ Although the frequency of PA in hypertensive patients is believed to be between 11% and 22%,^8^ only about 1%–4% of cases eligible for PA screening are tested.^9^, ^10^, ^11^

In PA, dysregulated production of aldosterone occurs despite suppression of plasma angiotensin and renin,^12^ which causes sodium retention and potassium wasting. This leads to hypokalaemia, increased extracellular water volume, elevated cardiac output, and increased arterial stiffness.^13^, ^14^, ^15^, ^16^ Hyperaldosteronism leads to deleterious effects on vascular and cardiac structures, including collagen deposition and CV fibrosis, endothelial dysfunction, perivascular inflammatory and fibrotic lesions, oxidative stress, and renal dysfunction.^15^ Compared with essential hypertension, PA is associated with a 2.3- to 4.0-fold risk of left ventricular (LV) hypertrophy, stroke, cardiac insufficiency, and atrial fibrillation, as shown by a meta-analysis of 31 trials.^15^

Since traditional haematoxylin-eosin (HE) staining does not reveal aldosterone synthase, a study of 2425 consecutive autopsies that found adrenal adenomas, nodules, or hyperplasia in 7% of hypertensive patients and in 1.9% of normotensive individuals did not establish a connection between these findings and adrenocortical hormonal excess.^17^ Recent histopathological methods have expanded the landscape of PA. Immunohistochemical staining for CYP11B2 (aldosterone synthase, cytochrome P450 family 11 subfamily B member 2) enables the histopathological classification of adrenocortical findings to aldosterone-producing adenomas (APA), aldosterone-producing nodules (APN), aldosterone-producing micronodules (APM), multiple APNs or APMs, aldosterone-producing diffuse hyperplasia, or aldosterone-producing adrenocortical carcinoma.^18^

Thus far, only case studies have linked PA to lethal arrhythmia.^19^ We hypothesized that adrenal causes, primarily PA, constitute an unrecognized risk for sudden death. Consequently, individuals succumbing to disease-induced sudden death (DSD) should exhibit more histopathologic findings consistent with autonomous aldosterone secretion in the adrenal glands than those who died from sudden trauma, suicide, or intoxication. We also searched for other adrenal risks for sudden death, including pheochromocytoma, as well as atrophy or bilateral adrenal metastases that might suggest the possibility of hypoadrenalism.

## Methods

### Study design and subjects

In this cross-sectional study, forensic pathologists examined 425 consecutive individuals who experienced sudden, unexpected deaths and were therefore subject to forensic investigation for the cause of death as required by law. In addition to the autopsy, the forensic determination of the cause of death involves microbiological, microscopic, toxicological, biochemical, and radiological investigations, when necessary, all aimed at identifying the cause of death. All deaths occurred in the southernmost region of Finland, including Helsinki and the counties of Uusimaa, Päijät-Häme, Kanta-Häme, Kymenlaakso, and South-Karelia. This region is home to approximately 2.4 million residents, or 43% of the Finnish population. The primary indications for forensic autopsy are shown in Supplementary Table S1 (Appendix p 8).

Data were collected from February 2022 to January 2023. Consecutive cases of sudden death were included based on the following criteria: individuals aged 35–70 years, with sudden unexpected death defined by one of the following conditions. 1) An unexpected, witnessed death occurring within 1 h of symptom onset in a person with or without previously known cardiac disease, with no identifiable extra-cardiac cause; 2) an unexpected, unwitnessed death occurring within the preceding 24 h, with no identifiable extra-cardiac cause; 3) death due to sudden trauma, suicide, or intoxication. Exclusion criteria included non-sudden death, terminal illness, or institutionalization of the patient, in order to balance age differences between cases and controls and to specifically target acute and sudden lethal events. This approach aimed to exclude deaths caused by infectious diseases or chronic diseases, such as malignancies and neurological conditions, which are not inherently associated with the cardiovascular risk characteristic of primary aldosteronism.

In our predefined preliminary immunohistochemical analysis, the refrigeration period and the maximum time in the refrigerator could be extended up to 168 (median 3.0) hours and 13 (median 7) days, respectively. Four individuals were excluded due to strong adrenal autolysis. Sixteen individuals were excluded due to missing adrenal samples, with one of them having only a unilateral adrenal sample. One individual was excluded due to uncertainty regarding the cause of death.

Routine autopsies were performed at the Department of Forensic Medicine, Helsinki. Data collected during autopsy included age, sex, time of death, cause of death, time from death to refrigerator, and refrigeration time. Sex was defined as female or male based on biological characteristics, as gender could not be reliably determined. Macroscopic and histologic findings of organs were collected.

This study complied with the Declaration of Helsinki and was approved by the Ethics Committee of the Helsinki and Uusimaa Hospital District (HUS/1646/2021) and Finnish Institute for Health and Welfare (THL/5008/5.05.00/2021). The study was registered at ClinicalTrials.gov (NCT05446779).

### Classification of sudden death

**Fig. 1:**
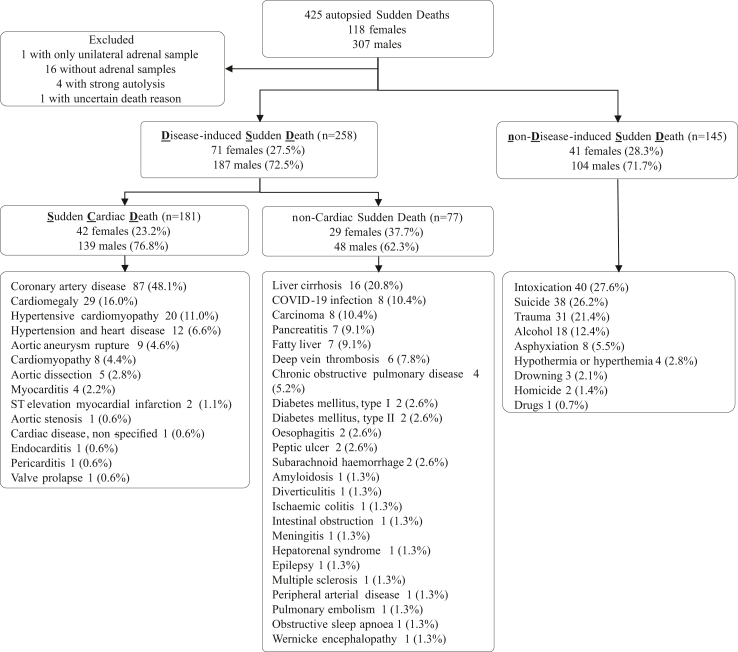
**Causes of death in the disease-induced sudden death (DSD) and non-disease-induced death (nDSD) groups.** The DSD group was reclassified into sudden cardiac death (SCD) and non-cardiac sudden death groups in post-hoc analyses.

Study subjects were classified into two groups after completion of all macroscopic, histopathological, biochemical, radiological, and toxicologic investigations related to the forensic autopsy: DSD and controls with non-disease-induced sudden death (nDSD), which includes deaths due to sudden trauma, suicide, or intoxication (Fig. 1). For post-hoc analyses, the DSD group was further divided into SCD and non-cardiac sudden death based on forensic autopsy findings that suggested either the presence or absence of major cardiac pathology as the cause of sudden death (Fig. 1, Supplementary Table S1, Appendix p 8).^2^^,^^20^

### Histopathology

In addition to standard operating procedures, paraffin-embedded tissue samples were prepared from both adrenal glands. All adrenals were cut into 3-mm thick slices. If the surface of the slices was visually intact, two separate slices were selected. If any abnormalities were suspected, three slices, preferably from areas with visual irregularities, were selected. These tissue slices were then formalin-fixed and paraffin-embedded for subsequent CYP11B2 staining. Three, two, or one stained sections of the right and left adrenals were available from 148, 241, or 14 individuals, and 190, 198, or 15 individuals, respectively. Histopathological analysis included the interpretation of haematoxylin and eosin (HE) staining and immunohistochemical staining with CYP11B2. Blinded adrenal histopathological examination was performed by a single pathologist with special expertise in endocrine pathology (HL). Immunohistochemical staining protocol and scoring of the stained slides have been described previously.^21^^,^^22^

**Fig. 2:**
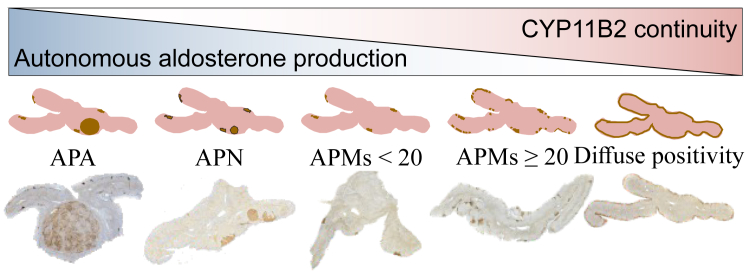
**Histopathological classification of CYP11B2 positivity**. This figure depicts the reciprocal relationship between the decrease in CYP11B2 continuity and the increase in autonomous aldosterone production, illustrated with examples of histopathological samples.

**Fig. 3:**
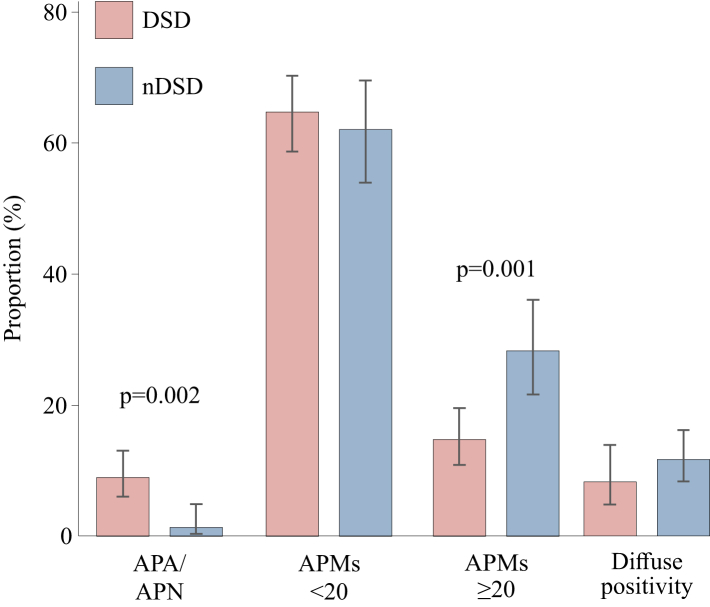
**Findings of CYP11B2 positivity**. The proportions of the CYP11B2 classification groups differed significantly between the disease-induced sudden death (DSD) and the non-disease-induced sudden death (nDSD) groups (p < 0.001). The prevalence of aldosterone-producing adenoma or aldosterone-producing nodules (APA/APN) was higher in the DSD group than in the nDSD group. A low number of aldosterone-producing micronodules (APMs <20) was detected more often in the DSD group, whereas APMs ≥20 and diffuse CYP11B2 positivity were found more frequently in the nDSD group. Data are mean with 95% confidence intervals (whiskers).

Histopathological analysis followed the HISTALDO consensus.^18^ The number of APMs in each slide was counted up to 19; counts of 20 or more were classified as APMs ≥20. A finding of CYP11B2 positivity in the zona glomerulosa (ZG) of ≥50% was classified as diffuse CYP11B2 positivity. A four-grade classification of CYP11B2 positivity was used in the post-hoc analyses with the following categories:1) APA/APN, 2) APMs <20, 3) APMs ≥20, or 4) diffuse CYP11B2 positivity. Each individual was categorized based on the lowest level of cortical CYP11B2 continuity found in any of the stained sections of each adrenal gland (Fig. 2, Fig. 3, Supplementary Figure S1 [Appendix p 12]).

### Statistical analysis

Sample size calculation is presented in the Supplementary Material (Appendix p 4). χ^2^ or Fisher's exact test was used for comparison of proportions of the categorical variables between the groups. Age, heart weight, and LV wall thickness were analysed using analysis of variance (ANOVA). The t-test was used for normally distributed variables, with means presented with standard deviation (SD) when appropriate. The Mann–Whitney *U* test was used as the nonparametric test for skewed variables, with results presented as median and interquartile range [IQR]. The Tukey's test was used in post-hoc analyses. Spearman's correlations with 95% confidence intervals (CI) were calculated for non-normally distributed variables. Binary logistic regression analyses were used to find explanatory factors for DSD and SCD.

Autopsy findings of myocardial infarction, LV hypertrophy, moderate or severe coronary artery disease (CAD), aortic atherosclerosis, peripheral vascular disease, cerebrovascular occlusion, and cardiac fibrosis were recorded to indicate presence of CV disease. The explanatory factors for the logistic regression analyses were age and autopsy-identified risk factors for CV death (heart weight, APA/APN, APMs <20, moderate or severe CAD, and myocardial fibrosis) (Supplementary Appendix p 4).

All tests were two-sided. Data analysis was conducted using SPSS software version 29.0 (IBM Corp. Armonk, NY, USA). P < 0.05 was considered statistically significant.

### Role of the funding source

The study was funded by independent grants from the Finnish State Research funds and three Finnish foundations. The sponsors had no role in study design; in the collection, analysis, or interpretation of data; in the writing of the report; or in the decision to submit the manuscript for publication.

## Results

### Causes of death

The DSD group included 258 individuals, while the nDSD control group included 145 individuals. The causes of death are shown in Fig. 1. No cases with sudden arrhythmic cardiac death syndrome with negative autopsy analysis were identified. Patient characteristics, including the findings indicating CV pathology, are presented in Table 1. None of the individuals had a prior record of adrenal disease.

**Table 1:** Patient characteristics in the disease-induced and non-disease-induced sudden death groups.

	Disease-induced sudden death	Non-disease-induced sudden death	p-value
Sex, n (%)			0.781
Male	187 (72.5)	104 (71.7)	
Female	71 (27.5)	41 (28.3)	
Age, y			
Females	58.9 (9.1)	55.2 (10.4)	0.055
Males	58.4 (8.9)	53.1 (10.9)	<0.001
BMI, kg/m^2^			
Females	28.5 (8.6)	29.4 (7.9)	0.612
Males	29.1 (7.5)	27.3 (6.4)	0.033
Autopsy findings			
Heart weight, g			
Females	435.0 (107.0)	382.0 (114.1)	0.017
Males	528.0 (132.6)	439.5 (105.2)	<0.001
Left ventricular wall, mm			
Females	13.4 (2.6)	12.8 (2.3)	0.281
Males	15.4 (3.1)	14.0 (2.8)	0.002
Right ventricular wall, mm			
Females	3.9 (1.1)	3.5 (0.7)	0.063
Males	4.2 (1.6)	3.7 (1.2)	0.025
Myocardial infarction^a^, n (%)	41 (15.9)	2 (1.4)	<0.001
Moderate or severe CAD, n (%)	114 (44.2)	23 (16.0)	<0.001
Peripheral arterial disease^b^, n (%)	104 (40.5)	38 (26.2)	0.004
Atherosclerosis in the aorta^b^, n (%)	177 (68.9)	68 (46.9)	<0.001
Cerebrovascular occlusion^b^, n (%)	57 (22.1)	23 (16.1)	0.149
Stroke, n (%)	2 (0.8)	0 (0)	0.539
Cardiac fibrosis, n (%)	134 (52.1)	48 (33.1)	<0.001
Kidney fibrosis, n (%)	111 (59.7)	55 (47.8)	0.045

Data are n (%) or mean (SD).

BMI, body mass index; CAD, coronary artery disease; SD, standard deviation.

a Including both acute and chronic.

b Including findings from mild to severe atherosclerosis.

### Histopathological findings with CYP11B2 immunohistochemistry

Adrenal histopathology revealed diagnostic changes in 7.7% of the cohort. Of these, the most prevalent (6.2%) were APA or APN (APA/APN). Among the whole cohort, one individual had APA (0.2%) positive for CYP11B2, and 24 individuals had APN (6.0%) (Fig. 3). APA/APN were detected in 3 (2.7%) females and 22 (7.6%) males. Three (0.7%) individuals had bilateral APNs (3 and 1 APNs, 2 and 2, 1 and 1 in the right and left adrenals, respectively). The mean age of individuals with APA/APN was 57.0 (SD 9.7) years, which was similar to the age of those without APA/APN at 56.8 years (SD 9.9).

Individuals with APA/APN (n = 25) were more frequently diagnosed with myocardial infarction (24.0% vs. 9.8%, p = 0.026) and exhibited a higher prevalence of atherosclerosis in the aorta (80.0% vs. 59.7%, p = 0.044) compared with those without APA/APN (n = 378) in age-adjusted analyses. The causes of death in individuals with APA/APN are presented in Supplementary Table S2 (Appendix p 9).

### Comparisons of CYP11B2 immunohistochemistry in subgroups

For the prespecified primary comparison, we analysed CYP11B2 findings between the DSD and nDSD groups. We found a significantly higher prevalence of APA/APN in the DSD group than in the nDSD group (8.9% vs. 1.4%, p = 0.002, Fig. 3). The detailed findings of CV pathology for these groups are shown in Supplementary Table S3 (Appendix p 10). Within the DSD group, the prevalence of APA/APN in the SCD and non-cardiac sudden death groups did not differ (8.8% and 9.1%, respectively).

The prevalence of APMs ≥20 was higher in nDSD group than in the DSD group (28.3% vs. 14.7%, p = 0.001, Fig. 3). The occurrence of APMs <20 was not significantly different between DSD and nDSD groups (64.7% vs. 62.1%, Fig. 3). None of the adrenal samples revealed CYP11B2-positive hyperplastic ZG cells (defined as diffuse hyperplasia according to HISTALDO).^18^

### Explanatory factors for disease-induced and sudden cardiac death in multivariable analyses

Explanatory factors for DSD were the presence of APA/APN, moderate or severe CAD, and heart weight. Also, the analysis of CYP11B2 continuity *per se* was a determinant of both DSD and SCD (Table 2). In males (n = 187), the explanatory factors for DSD were the presence of APA/APN (5.55 [95% CI 1.12–27.48], p = 0.036), moderate or severe CAD (4.95 [95% CI 2.43–10.07], p < 0.001), and heart weight (1.07 [95% CI 1.04–1.11], p < 0.001). In contrast, none of the above factors were significant explanatory factors among females (n = 71).

**Table 2:** Explanatory factors for disease-induced sudden death (DSD) and sudden cardiac death (SCD).

	Exposed (n)		Disease-induced sudden death					
			Univariate		p-value	Multivariable		p-value
	DSD	nDSD	OR	95% CI		OR	95% CI	
CYP11B2 continuity					0.009			0.027
Diffuse positivity or APMs ≥20	68	53	1			1		
APMs <20	167	90	1.45	0.93–2.25	0.101	0.84	0.50–1.39	0.489
APA/APN	23	2	8.96	2.02–39.75	0.004	6.47	1.40–29.88	0.017
Moderate or severe CAD	114	23	4.17	2.50–6.93	<0.001	3.34	1.88–5.93	<0.001
Heart weight (10 g)^a^	–	–	1.06	1.04–1.08	<0.001	1.05	1.03–1.08	<0.001
Age	–	–	1.05	1.03–1.07	<0.001	1.02	1.00–1.05	0.066
Myocardial fibrosis	134	48	2.20	1.44–3.36	<0.001	1.22	0.75–1.98	0.428

	Exposed (n)		Sudden cardiac death					
			Univariate		p-value	Multivariable		p-value
	SCD	nDSD	OR	95% CI		OR	95% CI	
CYP11B2 continuity					<0.001			0.020
Diffuse positivity or APMs ≥20	36	53	1			1		
APMs <20	129	90	2.11	1.28–3.49	0.004	1.30	0.69–2.42	0.417
APA/APN	16	2	11.78	2.55–54.38	0.002	10.68	2.02–56.43	0.005
Moderate or severe CAD	100	23	6.50	3.81–11.07	<0.001	6.54	3.42–12.50	<0.001
Heart weight (10 g)^a^	–	–	1.08	1.06–1.11	<0.001	1.08	1.06–1.11	<0.001
Myocardial fibrosis	104	48	2.77	1.75–4.36	<0.001	1.60	0.91–2.81	0.101
Age	–	–	1.06	1.04–1.09	<0.001	1.02	0.98–1.05	0.281

For DSD, the analysis included the entire study population (DSD group, n = 258; non-Disease-induced sudden death (nDSD) group, n = 145). For SCD, the analysis included the SCD group (n = 181) and the nDSD group (n = 145). The tested explanatory variables were age, sex, heart weight, adrenal histopathology consistent with APA/APN or APMs <20, moderate or severe coronary artery disease (CAD), and myocardial fibrosis.

Three individuals were excluded from the logistic regression analyses due to missing data.

APA, aldosterone-producing adenoma; APM, aldosterone-producing micronodule; APN, aldosterone-producing nodule; CAD, coronary artery disease; CI, confidence interval.

a Expressed per 10 g of heart mass.

When we included the subgroups of SCD (n = 181) and nDSD (n = 145) in the logistic regression analysis, the explanatory factors for SCD were APA/APN, moderate or severe CAD, and heart weight, while age was not an explanatory factor (Table 2). In females, moderate or severe CAD and heart weight were explanatory factors for SCD (OR 4.12 [95% CI 1.29–13.19], p = 0.017 and 1.06 [95% CI 1.01–1.12], p = 0.023, respectively). In males, explanatory factors for SCD were APA/APN (OR 7.12 [95% CI 1.12–45.20], p = 0.043), moderate or severe CAD (8.54 [95% CI 3.86–18.89], p < 0.001), and heart weight (1.10 [95% CI 1.07–1.14], p < 0.001).

### CYP11B2-continuity and findings of CV pathology

All individuals were classified into a four-grade system of decreasing ZG CYP11B2 continuity as shown in Supplementary Figure S1 (Appendix p 12), with the following categories:1) APA/APN, 2) APMs <20, 3) APMs ≥20, or 4) diffuse CYP11B2 positivity (Figs. 2 and 3). In addition to APA/APN in 25 (6.2%), APMs <20 were found in 257 (63.8%), and APMs ≥20 in 79 (19.6%) individuals. Symmetrical diffuse CYP11B2 positivity was found in 42 (10.4%) individuals. Fig. 3 shows the distribution of these categories and Supplementary Figure S2 (Appendix p 13) the symmetry of the findings.

**Fig. 4:**
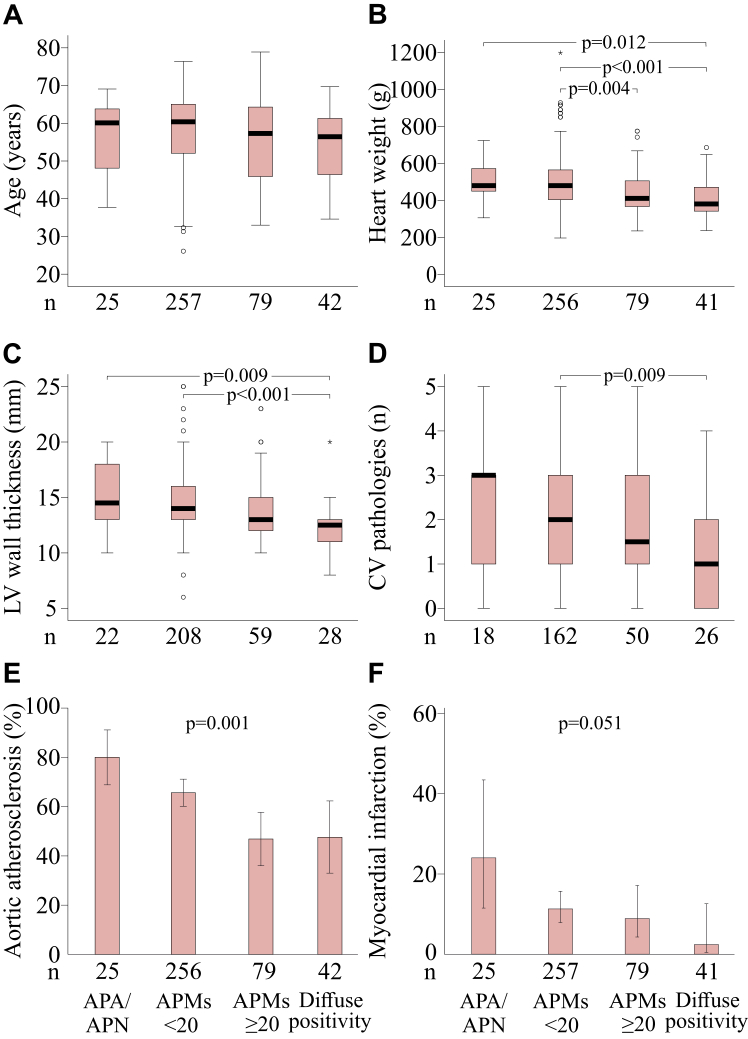
**Comparison of CYP11B2-continuity groups in the whole study population**. Age (A), heart weight (B), left ventricular wall (LV) thickness (C), number of cardiovascular (CV) pathologies (D), prevalence of aortic atherosclerosis (E), and prevalence of myocardial infarction (F). The CV pathologies that were identified in autopsy were moderate or severe coronary artery disease, aortic atherosclerosis, cerebrovascular occlusion, and heart fibrosis. Data are median (thick line), interquartile range (box), and range (whiskers) in panels A–D, and mean with 95% confidence intervals (whiskers) in panels E and F.

We then evaluated the associations between the classification of CYP11B2 continuity and findings of CV pathology at autopsy. All signs of CV pathology showed an increasing trend with decreasing CYP11B2 continuity (Fig. 4; ANOVA and pairwise comparisons).

### Additional adrenal histopathological findings

Adrenal histopathology revealed changes not associated with aldosterone production in 1.5% of the cohort. These changes were all found in the DSD group and included two pheochromocytomas with diameters of 7 mm and 12 mm, observed in individuals with hypertension and diabetes. The individual with a 7-mm pheochromocytoma suffered from CAD with acute myocardial infarction and also had prominent contralateral adrenal medulla, suggesting the possibility of MEN2 syndrome. The other individual had cardiomegaly and signs of alcohol dependence (Supplementary Table S2, Appendix p 9). Three individuals had adrenal metastatic lesions (Appendix p 6). One individual presented with partial cortical atrophy.

## Discussion

Sudden death is a high-priority public health concern, affecting at least 249,500 individuals each year in the European Union.^23^ We conducted the cross-sectional study of bilateral adrenal histopathology in 425 individuals with no history of adrenal disease who died suddenly. The study included both disease-induced deaths and those who died suddenly from other causes as control individuals. We observed that adrenal histopathology suggesting autonomous aldosterone production was found 6.4 times more frequently in individuals with DSD compared to those who died from non-disease causes. The presence of APA/APN and decrease of CYP11B2 continuity *per se* were associated with sudden death and were also independently related with risk factors for CV death. Therefore, our novel exploratory results raise the possibility that the presence of APA/APN contributes to sudden death. In general, primary aldosteronism can be easily screened for and effectively treated; however, whether this strategy can reduce the risk of sudden death over a lifetime warrants further investigation.

The present results suggest that adrenal histopathology consistent with autonomous aldosterone production represents an unrecognized cause of disease-driven sudden death; however, the findings obtained with adrenal CYP11B2 staining should be interpreted cautiously as an indicator of undiagnosed clinical primary aldosteronism. Yet, even the presence of adrenal APMs has been linked with aldosterone excess.^24^ We found that the presence of APA/APN was associated with higher rates of CV pathology at autopsy and was a strong independent explanatory factor for both DSD and SCD. Among those with DSD, the proportions of individuals with APA/APN did not differ between the SCD and non-cardiac sudden death groups (8.8% and 9.1%, respectively). One possible explanation is that the number of CV risk factors, including APA/APN, may have contributed to sudden death in individuals with a high disease burden in the non-cardiac sudden death group. It is feasible that histopathology consistent with PA could predispose individuals to hypokalaemia and fatal arrhythmia.^19^ However, plasma potassium concentrations in our cohort remain unknown.

We applied a classification based on CYP11B2 positivity and continuity to four groups: diffuse CYP11B2 positivity, APMs ≥20, APMs <20, and APA/APN. This classification reflects the decreasing continuity of ZG and increasing autonomous aldosterone production (Figs. 2 and 3).^25^ We found that diffuse symmetric CYP11B2 positivity (10.4% of individuals) and APMs ≥20 (19.6% of individuals) were not associated with significant CV pathology at autopsy or the risk of DSD. Previous postmortem studies focusing on age-related changes in the adrenal cortex are consistent with our findings that diffuse CYP11B2 positivity and numerous APMs are common and benign findings.^26^, ^27^, ^28^, ^29^, ^30^ It should be noted that we did not find a correlation between age and the number of APMs, contrary to some earlier studies.^16^^,^^28^^,^^30^

When compared with previous autopsy-based studies on adrenal pathology that examined immunohistochemical CYP11B2 staining, our study, which included over 400 deceased individuals, is larger.^17^^,^^26^, ^27^, ^28^^,^^30^ Moreover, our study was designed to link CV pathology at autopsy and the cause of death to adrenal histopathology. As discussed above, APA/APN were clearly related to DSD and various signs of CV pathology. In addition, among individuals with APA/APN and APMs <20, heart mass and LV wall thickness were the highest, and the number of CV pathologies (i.e., moderate or severe CAD, aortic atherosclerosis, cerebrovascular occlusion, and heart fibrosis) was the greatest when compared with individuals with diffuse CYP11B2 positivity or APMs ≥20. However, in a subgroup analysis, this finding applied only to males, likely due to the smaller sample size of females or poorly understood sex-specific differences in the pathophysiology of CV disease.^31^ Additionally, APMs <20 was a significant explanatory factor for SCD in age-adjusted univariate analysis. This novel finding suggests that, in addition to APA and APN, discontinuity of ZG defined by a low number of APMs is (APMs <20) is related to more severe CV pathology and an increased risk of sudden death.

The present findings emphasise the importance of APA/APN and decrease of CYP11B2 continuity in DSD cases; however, challenges in detecting these factors over a lifetime remain. The CYP11B2-positive APA/APN found in histological analyses were mostly too small to be detected by anatomical imaging and may therefore represent silent killers only detectable through biochemical testing. The ‘subclinical’ nature of CYP11B2 positivity in our study participants, or the global underdiagnosis of clinical PA,^9^^,^^11^ may explain the lack of identification of PA over a lifetime. It should be noted that the vast majority of PA is due to somatic mutations, which limits the effectiveness of genetic screening for PA.^16^ One way to improve diagnostics would be to make better use of electronic health record advisories to assist general practitioners with PA screening.^32^ This approach could alert doctors to potential cases and suggest appropriate tests, facilitating early detection and treatment of PA.^9^, ^10^, ^11^ Unilateral PA is surgically curable^33^ while mineralocorticoid receptor antagonists (MRAs) are highly effective in controlling hypokalaemia and improving outcomes in nonsurgical cases.^14^ Another way to improve outcomes is to advocate for more wide-ranging screening of PA in all hypertensive patients according to the European Society of Cardiology 2024 guideline, and more prevalent use of MRAs.^34^^,^^35^

The opportunity to focus on CYP11B2 examination of both adrenal glands offers novel insights into adrenal histopathology. The present analyses indicated autonomous aldosterone production in 6.2% of the total cohort. Interestingly, only 12.0% of individuals with APA/APN had symmetrical findings in adrenal immunohistochemistry, which is significantly lower than the up to 60% prevalence of bilateral aldosterone excess reported in clinically diagnosed PA.^16^ It is also noteworthy that the present APMs <20 group showed an increased number of CV pathologies. Large incidentalomas in the adrenals are frequently detected using modern anatomical imaging techniques, but these were not linked with sudden death in the present study (data not shown).

Pheochromocytomas may remain subclinical or be misdiagnosed until sudden death occurs. The prevalence of pheochromocytomas in autopsy series is approximately 0.05%.^36^ Notably, the two cases of pheochromocytoma were found in the DSD group, suggesting their potential contribution to sudden death. We did not identify any individuals with adrenocortical insufficiency, which is also known to increase mortality. Adrenal metastases were rare, occurring in only three individuals.

The participants could not be matched because the DSD and non-DSD groups were classified based on extensive and time-consuming forensic autopsy investigations. However, the consecutive collection of SD subjects helped to limit sampling bias. In addition to the study population size, our study design also limited the possibility to link the presence of APA/APN to hypertension. The cohort included individuals with hypertension, those without hypertension, and individuals whose blood pressure was unknown. However, a significant correlation between LV mass/heart weight and progressive histopathological findings consistent with PA suggests an association with hypertension. To minimize potential bias related to age and sex between the DSD and nDSD groups, the inclusion criteria were adjusted to target individuals older than 35 years of age. We did not have access to all information in the participants’ lifetime medical records or medication histories. Therefore, we lack information on several cardiovascular risk factors, such as the use of blood pressure lowering medications, statins, aspirin, as well as data on socioeconomic status, diabetes status, kidney function, and similar factors. Such information could have provided new insights into the associations between adrenal histology and morbidity. More research is needed to evaluate whether a subpopulation of APMs could be considered clinically significant precursors of APAs and whether they could be related to diagnosable clinical primary aldosteronism.^24^

A strength of our study was the thorough examination of causes of death by forensic pathologists and the detailed bilateral analysis of adrenal CYP11B2 by an experienced endocrinological pathologist, following the consensus classification principles.^18^ The selection of adrenal blocks was based on visual inspection during autopsy, and the analysis included one to three sections from both adrenal glands. Nevertheless, this approach may have missed some microscopic cortical foci. Surprisingly, none of the samples showed diffuse hyperplasia as defined by the consensus. One possible reason is that HISTALDO is based on immunohistochemical staining of only 37 patients with clinically diagnosed unilateral PA.^18^

### Conclusions

Unexpected sudden death particularly shortens the lifespan of young and middle-aged individuals during their productive years. Therefore, novel, preventive strategies are urgently needed.^7^ Our study provided a unique opportunity to link the spectrum of adrenal histopathology to the cause of death and to compare these findings with those whose deaths were not related to adrenal pathology or CV causes. We found a considerable number of adrenal pathologies suggesting autonomous aldosterone production and two cases of pheochromocytoma in our cohort of sudden death. Among those with DSD, APA/APN and loss of continuity in CYP11B2 expression in the adrenal zone glomerulosa were strongly associated with findings of significant CV pathology. Most importantly, staining with CYP11B2 revealed APA/APN in 8.9% of cases with DSD.

Taken together, our results suggest that the presence of APA/APN and a decrease of CYP11B2 continuity suggesting autonomous aldosterone production may be a novel risk factor for sudden death. Timely diagnosis and effective treatment of PA may extend the lifespan of individuals with CYP11B2 positive adrenal pathology. Whether systematic biochemical screening for PA, targeted MRA therapy, or both should be adopted to reduce the risk of sudden death remains to be studied.

## Contributors

NM, PIN, HL, TV, and AV conceptualised and designed the study. AV, HL, and TV conducted the analyses. AY, JI, HH, NM, and PIN had access to raw data and verified the data in the manuscript, and AY, JI, and HH undertook data analysis. AY, JI, ALA, MV, EK, IP, NM, and PIN interpreted the data. AY, NM, and PIN drafted the manuscript. All authors actively participated in finalising and approving the manuscript. PIN and NM had final responsibility for the decision to submit for publication.

## Data sharing statement

The datasets generated and analysed during the current study are not publicly available due to national laws on data protection. We will consider sharing deidentified, individual participant level data that underlie the results reported in this article on receipt of a request detailing the study hypothesis and statistical analysis plan. All requests should be sent to the corresponding author. The corresponding author and lead investigators of this study will discuss all requests with the authorities at the Finnish Institute for Health and Welfare and make decisions about whether data sharing is appropriate based on EU legislation and the scientific rigor of the proposal. All applicants will be asked to sign a data access agreement.

## Declaration of interests

The authors declare no conflicts of interest with respect to this manuscript.
