# Supplementary material for: Adrenal aldosterone synthase (CYP11B2) histopathology and its association with disease-induced sudden death: a cross-sectional study
Source: Lancet Reg Health Eur. 2025 Feb 6;51:101226. doi: 10.1016/j.lanepe.2025.101226 (PMC11849129; doi:10.1016/j.lanepe.2025.101226)
Supplement: Supplementary Figures and Tables [file mmc1.docx]

**Supplementary appendix**

Supplement to:

Ylänen A, Isojärvi J, Virtanen A, et al. **Adrenal aldosterone synthase (CYP11B2) histopathology and its association with disease-induced sudden death: a cross-sectional study.**

**CONTENTS**

Cortical CYP11B2-continuity classification

Statistical analysis

Supplementary results

References

Supplementary table 1

Supplementary table 2

Supplementary table 3

Supplementary figure 1

Supplementary figure 2

STROBE checklist

**Abbreviations:** APA, aldosterone-producing adenoma; APM, aldosterone-producing micronodule; APN, aldosterone-producing nodule; CACNA1D, somatic mutation encoding the α1 subunit of the L-type voltage-dependent calcium channel Cav1.3; CAD, coronary artery disease; CV, cardiovascular; CYP11B2, cytochrome P450 family 11 subfamily B member 2; DSD, disease-induced sudden death; HE, haematoxylin-eosin; IQR, interquartile range; LV, left ventricular; nDSD, non-disease-induced sudden death; PA, primary aldosteronism; SCD, sudden cardiac death; SD, standard deviation; ZG, zona glomerulosa

**Cortical CYP11B2-continuity classification**

We divided the adrenal findings into a four-grade classification according to CYP11B2 positivity. The categories were 1) aldosterone-producing adenoma or aldosterone-producing nodule (APA/APN), 2) aldosterone-producing micronodule (APMs) <20, 3) APMs ≥20, or 4) diffuse CYP11B2 positivity, which were used in post-hoc analyses as illustrated in Supplementary Figure 1. The classification to those with <20 or ≥20 APMs was based on previous data showing that a unique feature of human zona glomerulosa (ZG) is its sharp demarcation between foci of dense CYP11B2 expression, which form APMs. However, CYP11B2 is paradoxically switched off in most ZG cells. More continuous expression is found mostly among young individuals or those without primary aldosteronism (PA).^1–4^ Autopsy findings from 83 adrenal glands of individuals up to 40 years of age revealed that the decline in the relative CYP11B2-expressing ZG area and continuity with age were indicative of an increase in clustering, comparable to the formation of APMs. The similarities in both adrenals from the same individual indicated that these changes occurred symmetrically.^2^ The study by Omata et al.^5^ suggests that these APMs are frequent in nonhypertensive adrenals, accumulate with age, and frequently harbour somatic mutations (most commonly in CACNA1D), which cause autonomous aldosterone production by increasing intracellular calcium.^6^ Further molecular genetic analyses support the concept that APMs serve as precursors of PA, as APMs were found to be common in normal adrenals and to harbour somatic mutations known to cause excess aldosterone production.^7^ The data therefore suggest that increasing discontinuity of ZG may precede the formation of APNs and APAs. It should be noted that most studies in individuals with PA lack some relevant clinical and biochemical data, and histological studies include only one adrenal per individual with PA.

**Statistical analysis**

Estimation of the sample size was based on assumptions of PA prevalence. Up to 5-22% of individuals with hypertension have biochemical hyperaldosteronism that remains mostly unrecognized.^8,9^ The prevalence of hypertension in the age group of 25-64 years is 52% in men and 34% in women.^10^ We expected the prevalence of histopathological PA to be approximately 12% in the disease-induced sudden death (DSD) group and approximately 3% in the non-disease-induced sudden death (nDSD) control group. With these assumptions, 154 individuals were required to be included in both groups (alpha=0·05 and power=0·80) to detect a significant difference in immunohistochemical PA occurrence.

Logistic regression analyses included the whole study population for the sudden death, the sudden cardiac death (SCD) subgroup, and the nDSD control group for SCD. The tested explanatory factors were age and autopsy-identified risk factors for cardiovascular (CV) death, including heart weight, APA/APN, APMs <20, moderate or severe coronary artery disease (CAD), and myocardial fibrosis. Moderate or severe CAD was selected to represent vascular complications in the logistic regression model, as CAD, aortic atherosclerosis, cerebrovascular disease, and peripheral artery disease are all manifestations of vascular morbidity and could introduce collinearity bias. Similarly, only heart weight, not left ventricular wall (LV) thickness, was included in the analyses.

The number of CV pathologies was based on the sum of findings of left ventricular hypertrophy (LVH, wall thickness ≥15 mm), moderate or severe CAD, aortic atherosclerosis, cerebrovascular disease, and cardiac fibrosis in the autopsy.

**Supplemental results**

*CYP11B2* *continuity in DSD vs. nDSD*

We evaluated the associations between the classification of CYP11B2 continuity and autopsy findings in the whole study population. Individuals with APA/APNs had a higher incidence of myocardial infarction with borderline significance compared to those with APMs <20, APMs ≥20, diffuse CYP11B2 positivity (24·0% vs. 11·3% vs. 8·9% vs. 2·4%, p=0·051, respectively). They also had higher prevalence of aortic atherosclerosis (80·0% vs. 65·6% vs. 46·8% vs. 47·6%, p=0·001). The cumulative number of CV pathologies in autopsy was lower in individuals with diffuse positivity than in individuals with APMs ≥20, APMs <20, or APA/APNs (median 1 [IQR 0-2] vs. 2 [1-3] vs. 2 [1-3] vs. 3 [1-3], p=0·006, respectively, figure 4 D). In a pairwise post-hoc test, the difference between diffuse positivity and APMs <20 and diffuse positivity and APA/APN remained significant (1 vs. 2, p=0·001) and (1 vs. 3, p=0.033), respectively. When heart weight was analysed with ANOVA using CYP11B2-continuity classification, individuals with APA/APNs had greater heart mass than those with APMs <20, APMs ≥20, or diffuse CYP11B2 positivity (503·2 vs. 494·1 vs. 437·8 vs. 404·5 g, p<0·001, respectively). Also, the age-adjusted difference in heart weight was significant between the diffuse positivity, APMs ≥20, and APMs <20 groups. In pairwise analyses, heart mass differed significantly between diffuse positivity and APMs <20 (404·5 vs. 494·1 g, p<0·001), between APMs <20 and APMs ≥20 (437·8 vs. 494·1 g, p<0.001), and diffuse positivity and APA/APNs (404·5 vs. 503·2 g, p<0·001). In analogy to heart mass, age-adjusted LV wall thickness was highest in individuals with APA/APNs when compared with APMs <20, APMs ≥20, or diffuse positivity (15·1 vs. 14·8 vs. 14·0 vs. 12·4 mm, p<0·001, respectively). In pairwise analyses, LV wall was significantly thicker in individuals with APA/APNs and APMs <20 compared with those with diffuse positivity (15·1 vs. 12·4 mm, p=0·001 and 14·8 vs. 12·4 mm, p<0·001, respectively).

*CYP11B2* *continuity in SCD vs. nDSD*

When the SCD group was compared with the nDSD group, the spectrum of CYP11B2 continuity differed significantly (diffuse positivity: 5·0% vs. 8·3%; APMs ≥20: 14·9% vs. 28·3%; APMs <20: 71·3% vs. 62·1%: and APA/APN: 8·8% vs. 1·4 %, respectively, p<0·001) (figure 1).

*Sex-specific classification according to CYP11B2 positivity*

The prevalence of diffuse CYP11B2 positivity was higher in females than in males (18·8% vs. 7·2%, p=0·001). There was no difference between females and males in the prevalence of APMs <20 or APMs ≥20 (data not shown).

To further dissect the significance of classification for CYP11B2 positivity, we performed sex-specific comparisons for age, heart weight, left ventricular wall (LV) thickness, and number of CV risk factors across the classification of CYP11B2 continuity between the DSD and nDSD groups. In both sexes, the groups with diffuse positivity had the lowest heart weight, LV wall thickness, and number of CV pathologies. In males, the results showed evidence of higher heart weight, increased LV wall thickness, and a greater number of CV pathologies when individuals in the APMs ≥20, APMs <20, and APA/APN groups were compared to those with diffuse CYP11B2 positivity (Supplementary figure 3). Males in the DSD group were significantly older than men in the nDSD group (58·5 vs. 53·1 years, p<0·001), but such differences were not found among females.

*Malignant adrenal* *histopathological findings in all individuals*

In the DSD group, two individuals had unilateral metastases: one due to a pulmonary or a thymic neuroendocrine carcinoma, and the other due to an adenocarcinoma of undetermined origin. One case of bilateral metastasis of breast cancer was found, but histopathology revealed sufficient preserved adrenal cortex to exclude the possibility of hypoadrenalism.

*Histopathological findings of the thyroid and pancreas in all individuals*

Autopsy findings of the thyroid and pancreas are shown in supplementary table 4.

**References**

1 Tezuka Y, Atsumi N, Blinder AR, *et al.* The age-dependent changes of the human adrenal cortical zones are not congruent. *J Clin Endocrinol Metab* 2021; **106**: 1389–97.

2 van de Wiel E, Chaman Baz A-H, Küsters B, *et al.* Changes of the CYP11B2 expressing zona glomerulosa in human adrenals from birth to 40 years of age. *Hypertension* 2022; **79**: 2565–72.

3 Hayashi T, Zhang Z, Al-Eyd G, *et al.* Expression of aldosterone synthase CYP11B2 was inversely correlated with longevity. *J Steroid Biochem Mol Biol* 2019; **191**: 105361.

4 Nanba K, Vaidya A, Williams GH, Zheng I, Else T, Rainey WE. Age-related autonomous aldosteronism. *Circulation* 2017; **136**: 347–55.

5 Omata K, Anand SK, Hovelson DH, *et al.* Aldosterone-producing cell clusters frequently harbor somatic mutations and accumulate with age in normal adrenals. *J Endocr Soc* 2017; **1**: 787–99.

6 Azizan EAB, Poulsen H, Tuluc P, *et al.* Somatic mutations in ATP1A1 and CACNA1D underlie a common subtype of adrenal hypertension. *Nat Genet* 2013; **45**: 1055–60.

7 Nishimoto K, Tomlins SA, Kuick R, *et al.* Aldosterone-stimulating somatic gene mutations are common in normal adrenal glands. *Proc Natl Acad Sci USA* 2015; **112**. DOI:10.1073/pnas.1505529112.

8 Morera J, Reznik Y. Management of endocrine disease: the role of confirmatory tests in the diagnosis of primary aldosteronism. *Eur J Endocrinol* 2019; **180**: R45–58.

9 Brown JM, Siddiqui M, Calhoun DA, *et al.* The unrecognized prevalence of primary aldosteronism: a cross-sectional study. *Ann Intern Med* 2020; **173**: 10–20.

10 Kastarinen M, Antikainen R, Peltonen M, *et al.* Prevalence, awareness and treatment of hypertension in Finland during 1982-2007. *J Hypertens* 2009; **27**: 1552–9.

***Supplementary table 1*:** Indications for autopsy in the study population of 425 consecutive individuals whose deaths were sudden and unexpected.

|  | **n (%)** |
| --- | --- |
| Unexpected sudden death | 313 (73·6) |
| Suicide | 54 (12·7) |
| Trauma | 34 (8·0) |
| Intoxication | 14 (3·3) |
| Trauma and intoxication | 4 (0·9) |
| Crime | 3 (0·7) |
| Intoxication and surgical treatment | 1 (0·2) |
| Surgical treatment | 1 (0·2) |
| Suicide and intoxication | 1 (0·2) |

***Supplementary table 2*:** Causes of death when a CYP11B2-positive adenoma (APA), nodule (APN), or pheochromocytoma (PHE) was identified.

| **N:o** | |  | | **Cause of death** | | **Contributing cause of death** | |
| --- | --- | --- | --- | --- | --- | --- | --- |
| Disease-induced sudden death | | | | | | | |
| 1 | | APA | | CAD | |  | |
| 2 | | APN | | CAD | | EH, and DM type II | |
| 3 | | APN | | CAD | | EH, and DM type II | |
| 4 | | APN | | CAD | |  | |
| 5 | | APN | | CAD | | Hyperlipidaemia | |
| 6 | | APN | | CAD | | Asthma | |
| 7 | | APN | | CAD | |  | |
| 8 | | APN | | CAD | |  | |
| 9 | | APN | | Cardiomegaly | | Obesity | |
| 10 | | APN | | Cardiomegaly | | Psychoactive substance abuse | |
| 11 | | APN | | Cardiomegaly | |  | |
| 12 | | APN | | Fatty liver, Alcoholic | | EH | |
| 13 | | APN | | Fatty liver, Alcoholic | | Acute alcohol intoxication | |
| 14 | | APN | | Fatty liver, Alcoholic | |  | |
| 15 | | APN | | EH and HF | | Acute alcohol intoxication | |
| 16 | | APN | | EH and HF | | Acute alcohol intoxication | |
| 17 | | APN | | EH and HF | | Obesity, opioid intoxication, and paranoid schizophrenia | |
| 18 | | APN | | EH and heart disease | |  | |
| 19 | | APN | | Aortic rupture | | EH | |
| 20 | | APN | | COPD | |  | |
| 21 | | APN | | DM, type I | | Cardiomegaly | |
| 22 | | APN | | DM, type II | | Fatty liver | |
| 23 | | APN | | COVID-19 infection | | DM type II | |
| 24 | | PHE | | Cardiomegaly | | Alcohol dependence | |
| 25 | | PHE | | CAD | | DM type II and EH | |
| non-Disease-induced sudden death | | | | | | | |
| 26 | | APN | | Suicide | | Acute alcohol intoxication and alcohol dependence | |
| 27 | | APN | | Hypothermia | | Acute alcohol intoxication | |

CAD, coronary artery disease; COPD, chronic obstructive pulmonary disease; COVID-19, coronavirus disease in 2019 caused by the SARS-CoV-2 virus; DM; diabetes mellitus; EH, essential hypertension; HF: heart failure

***Supplementary table 3*:** **Detailed autopsy findings in the disease-induced sudden death and non-disease-induced sudden death groups**.

|  | **Disease-induced sudden death** |  | **Non-disease-induced sudden death** | **p-value** |
| --- | --- | --- | --- | --- |
|  | n (%) |  | n (%) |  |
| Myocardial Infarction |  |  |  | <0·001 |
| No | 217 (84·1) |  | 142 (98·6) |  |
| Acute | 23 (8·9) |  | 0 (0) |  |
| Chronic | 18 (7·0) |  | 2 (1·4) |  |
| Coronary artery disease* |  |  |  | <0·001 |
| No | 77 (29·8) |  | 76 (52·8) |  |
| Mild | 67 (26·0) |  | 45 (31·3) |  |
| Moderate | 60 (23·3) |  | 16 (11·1) |  |
| Severe | 54 (20·9) |  | 7 (4·9) |  |
| Aortic valve abnormality | 12 (4·7) |  | 4 (2·8) | 0·435 |
| Other valve abnormality | 6 (2·3) |  | 3 (2·1) | 1·000 |
| Peripheral arterial disease* |  |  |  | 0·010 |
| No | 153 (59·5) |  | 107 (73·8) |  |
| Mild atherosclerosis | 86 (33·5) |  | 34 (23·4) |  |
| Severe atherosclerosis | 18 (7·0) |  | 4 (2·8) |  |
| Atherosclerosis in the aorta |  |  |  | <0·001 |
| No | 80 (31·1) |  | 77 (53·1) |  |
| Mild atherosclerosis | 139 (54·1) |  | 60 (41·4) |  |
| Severe atherosclerosis | 38 (14·8) |  | 8 (5·5) |  |
| Cerebrovascular occlusion |  |  |  | 0·272 |
| No | 201 (77·9) |  | 120 (83·9) |  |
| Mild atherosclerosis | 51 (19·8) |  | 22 (15·4) |  |
| Severe atherosclerosis | 6 (2·3) |  | 1 (0·7) |  |
| Kidney abnormality* |  |  |  | 0·006 |
| No | 154 (59·7) |  | 108 (75·0) |  |
| Mild changes | 99 (38.4) |  | 35 (24·3) |  |
| Abnormal | 5 (1·9) |  | 1 (0·7) |  |
| Liver |  |  |  | 0·443 |
| Normal | 81 (31·4) |  | 41 (28·3) |  |
| Fatty liver | 110 (42·6) |  | 78 (53·8) |  |
| Fibrosis | 3 (1·2) |  | 2 (1·4) |  |
| Cirrhosis | 9 (3·5) |  | 3 (2·1) |  |
| Fatty liver and fibrosis | 24 (9·3) |  | 9 (6·2) |  |
| Fatty liver and cirrhosis | 21 (8·1) |  | 6 (4·1) |  |
| Carcinoma | 3 (1·2) |  | 2 (1·4) |  |
| Other | 7 (2·7) |  | 4 (2·8) |  |
| Endocrinological abnormalities | 18 (7·0) |  | 9 (6·2) | 0·767 |

***Supplementary table 4*:** Autopsy findings in the thyroid and pancreas.

|  | **n (%)** |
| --- | --- |
| Thyroid |  |
| Nodularity | 5 (1·2) |
| Hyperplasia | 7 (1·7) |
| Cyst | 2 (0·5) |
| Atrophy | 1 (0·2) |
| Pancreas |  |
| Fibrosis | 5 (1·2) |
| Cyst | 2 (0·5) |
| Inflammation | 2 (0·5) |
| Other | 3 (0·7) |


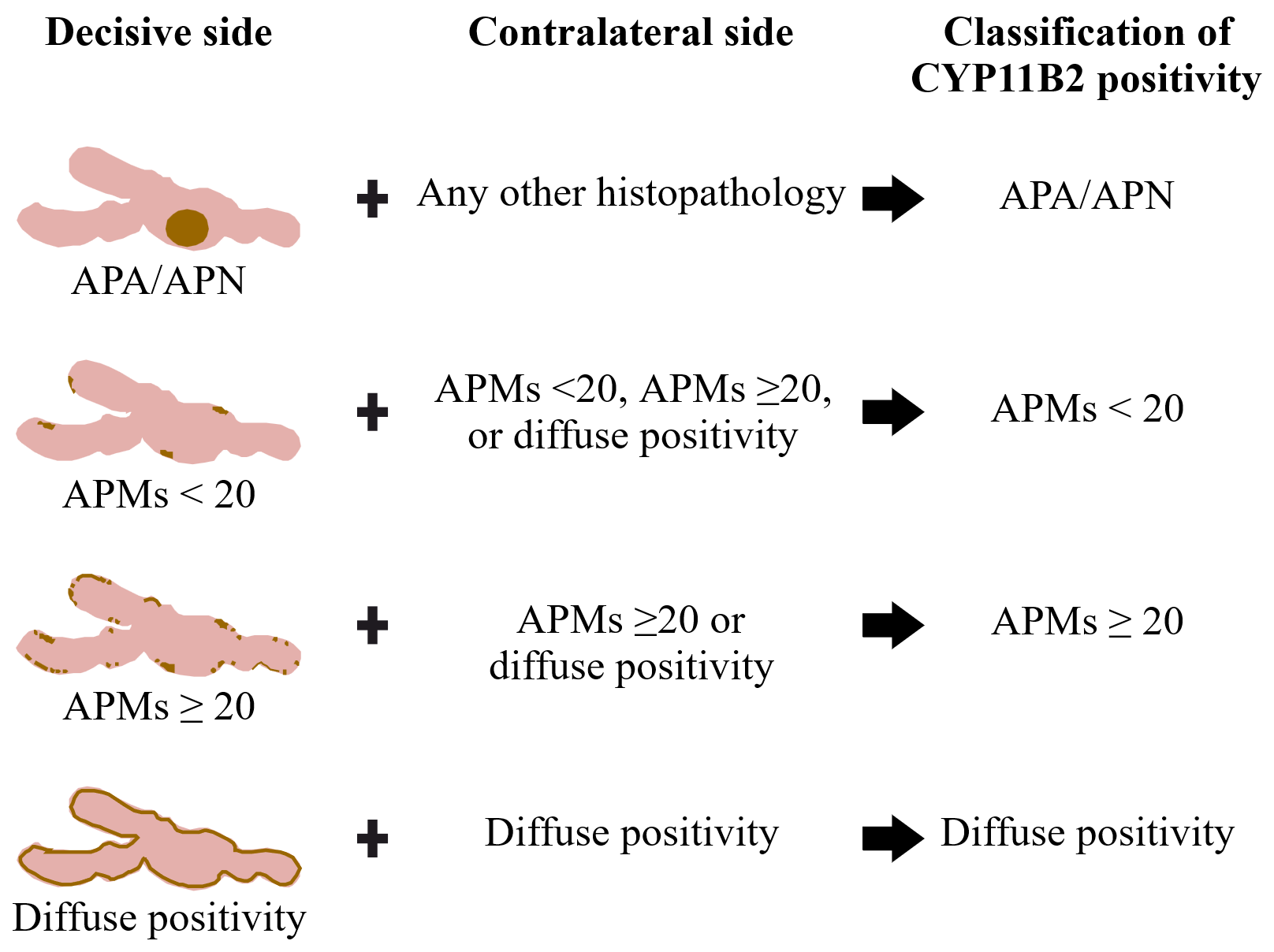


**Supplementary figure 1:** **Classification of CYP11B2 continuity**

The least continuous CYP11B2-positive staining observed in any section of either adrenal gland was used to determine the classification for the individual. Two or three stained tissue sections were available for classification from 777 adrenals and one section from 29 adrenals.


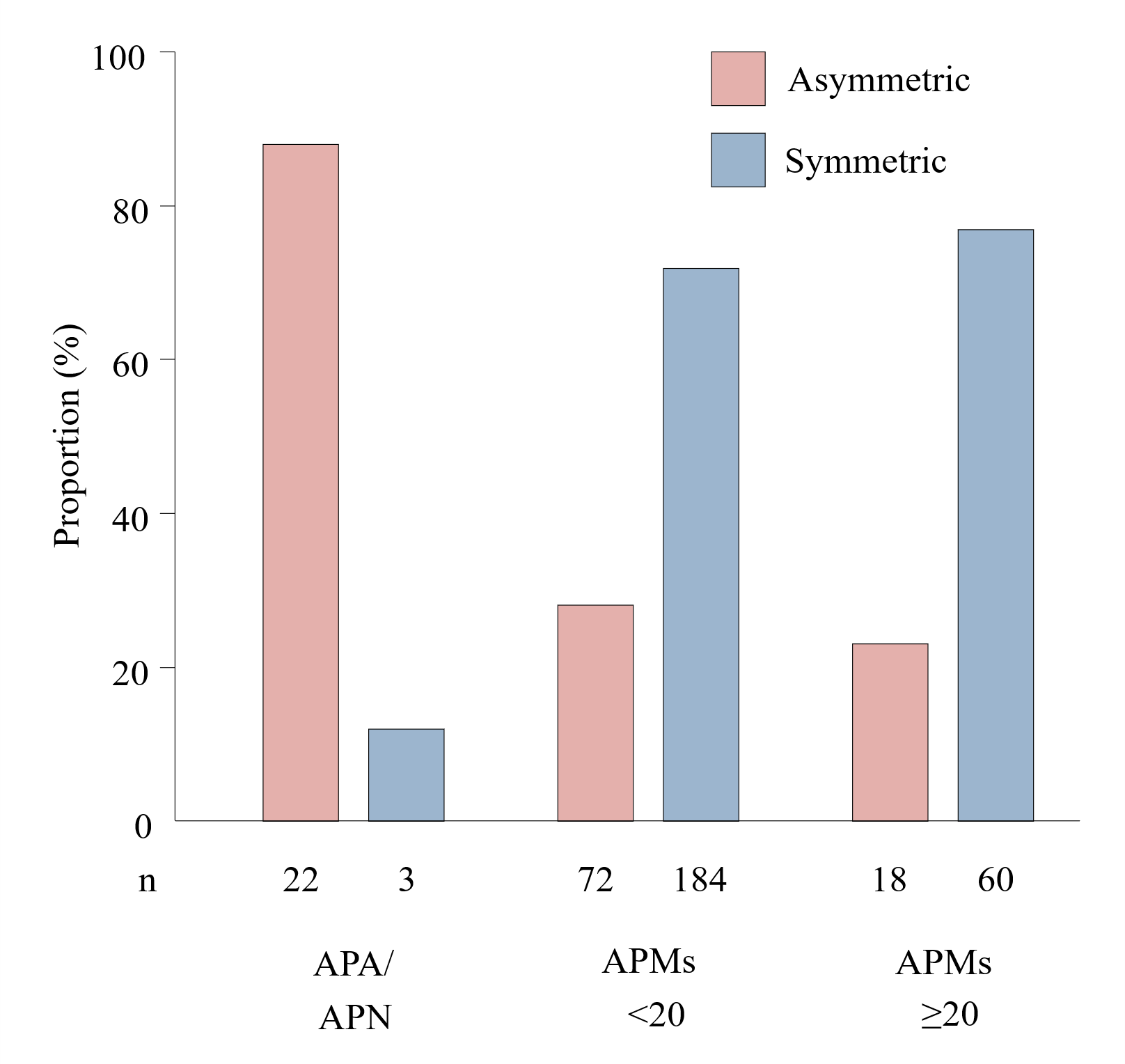


**Supplementary figure 2:** **Symmetry of CYP11B2 continuity categories**

Cases with concordant or discordant CYP11B2 continuity between the two adrenal glands were classified as symmetric or asymmetric, respectively. The classification was based on the lowest level of CYP11B2 continuity observed in either adrenal gland, as presented in supplementary figure 1.

**
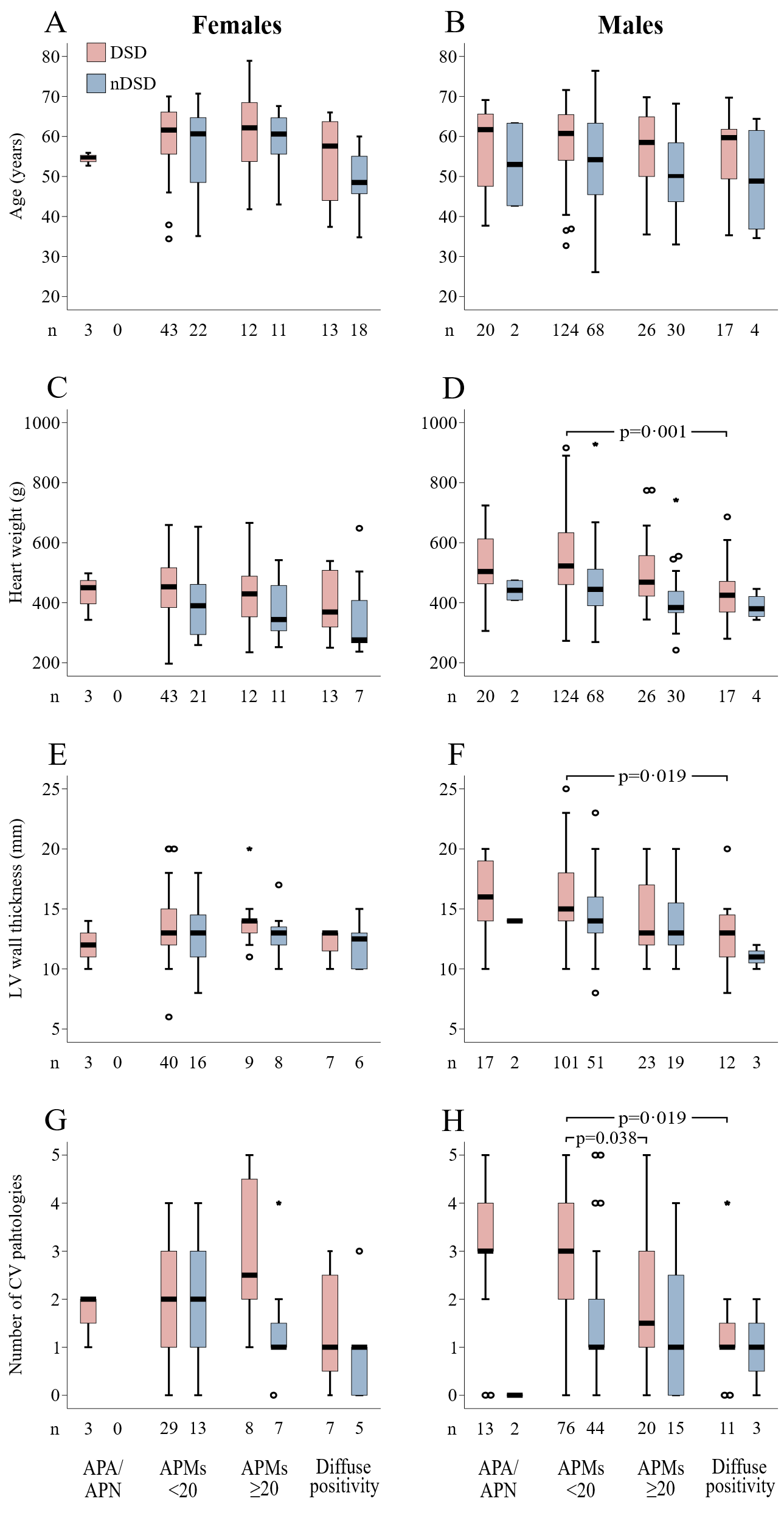
**

**Supplementary figure 3:** **Sex-specific results with CYP11B2** **classification**

In males, individuals with APA/APNs and APMs <20 showed a trend towards higher heart weight, increased left ventricular wall (LV) thickness, and a greater number of cardiovascular (CV) pathologies compared to those with APMs ≥20 and diffuse positivity. DSD, disease-induced sudden death; nDSD, non-disease-induced sudden death; median (thick line), 25^th^-75^th^ percentile (box), range (whiskers).

STROBE Statement—Checklist of items that should be included in reports of ***cross-sectional studies***

|  | Item No | Recommendation |  | Page |  |
| --- | --- | --- | --- | --- | --- |
| **Title and abstract** | 1 | (*a*) Indicate the study’s design with a commonly used term in the title or the abstract |  | 1 |  |
|  |  | (*b*) Provide in the abstract an informative and balanced summary of what was done and what was found |  | 2 |  |
| Introduction | | |  |  |  |
| Background/rationale | 2 | Explain the scientific background and rationale for the investigation being reported |  | 6-7 |  |
| Objectives | 3 | State specific objectives, including any prespecified hypotheses |  | 7 |  |
| Methods | | |  |  |  |
| Study design | 4 | Present key elements of study design early in the paper |  | 7-9 |  |
| Setting | 5 | Describe the setting, locations, and relevant dates, including periods of recruitment, exposure, follow-up, and data collection |  | 7-9 |  |
| Participants | 6 | (*a*) Give the eligibility criteria, and the sources and methods of selection of participants |  | 7-8 |  |
| Variables | 7 | Clearly define all outcomes, exposures, predictors, potential confounders, and effect modifiers. Give diagnostic criteria, if applicable |  | 9-10 |  |
| Data sources/ measurement | 8* | For each variable of interest, give sources of data and details of methods of assessment (measurement). Describe comparability of assessment methods if there is more than one group |  | na |  |
| Bias | 9 | Describe any efforts to address potential sources of bias |  | 8 |  |
| Study size | 10 | Explain how the study size was arrived at |  | 10 |  |
| Quantitative variables | 11 | Explain how quantitative variables were handled in the analyses. If applicable, describe which groupings were chosen and why |  | 9-10 |  |
| Statistical methods | 12 | (*a*) Describe all statistical methods, including those used to control for confounding |  | 10 and Suppl 4 |  |
|  |  | (*b*) Describe any methods used to examine subgroups and interactions |  | 9 and Suppl 4 |  |
|  |  | (*c*) Explain how missing data were addressed |  | na |  |
|  |  | (*d*) If applicable, describe analytical methods taking account of sampling strategy |  | na |  |
|  |  | (*e*) Describe any sensitivity analyses |  | na |  |
| Results | | |  |  |  |
| Participants | 13* | (a) Report numbers of individuals at each stage of study—eg numbers potentially eligible, examined for eligibility, confirmed eligible, included in the study, completing follow-up, and analysed |  | 11 |  |
|  |  | (b) Give reasons for non-participation at each stage |  | na |  |
|  |  | (c) Consider use of a flow diagram |  | na |  |
| Descriptive data | 14* | (a) Give characteristics of study participants (eg demographic, clinical, social) and information on exposures and potential confounders |  | 9 and Table 1 |  |
|  |  | (b) Indicate number of participants with missing data for each variable of interest |  | na |  |
| Outcome data | 15* | Report numbers of outcome events or summary measures |  | 11-13 |  |
| Main results | 16 | (*a*) Give unadjusted estimates and, if applicable, confounder-adjusted estimates and their precision (eg, 95% confidence interval). Make clear which confounders were adjusted for and why they were included |  | 12-13, Table 2, FigS3 |  |
|  |  | (*b*) Report category boundaries when continuous variables were categorized |  | 12-13 |  |
|  |  | (*c*) If relevant, consider translating estimates of relative risk into absolute risk for a meaningful time period |  | na |  |
| Other analyses | 17 | Report other analyses done—eg analyses of subgroups and interactions, and sensitivity analyses |  | Suppl 4 |  |
| Discussion | | |  |  |  |
| Key results | 18 | Summarise key results with reference to study objectives |  | 4, 14,18 |  |
| Limitations | 19 | Discuss limitations of the study, taking into account sources of potential bias or imprecision. Discuss both direction and magnitude of any potential bias |  | 17-18 |  |
| Interpretation | 20 | Give a cautious overall interpretation of results considering objectives, limitations, multiplicity of analyses, results from similar studies, and other relevant evidence |  | 14-18 |  |
| Generalisability | 21 | Discuss the generalisability (external validity) of the study results |  | 14 |  |
| Other information | | |  |  |  |
| Funding | 22 | Give the source of funding and the role of the funders for the present study and, if applicable, for the original study on which the present article is based |  | 3,11,19 |  |

*Give information separately for exposed and unexposed groups.

**Note:** An Explanation and Elaboration article discusses each checklist item and gives methodological background and published examples of transparent reporting. The STROBE checklist is best used in conjunction with this article (freely available on the Web sites of PLoS Medicine at http://www.plosmedicine.org/, Annals of Internal Medicine at http://www.annals.org/, and Epidemiology at http://www.epidem.com/). Information on the STROBE Initiative is available at www.strobe-statement.org.
